# Postictal punctate hippocampal diffusion restriction: the chicken or the egg?

**DOI:** 10.3389/fneur.2025.1659610

**Published:** 2025-09-29

**Authors:** Jan Heckelmann, Yvonne Weber, Manuel Dafotakis, Stefan Wolking

**Affiliations:** ^1^Department of Epileptology and Neurology, RWTH University Hospital Aachen, Aachen, Germany; ^2^Department of Neurology, RWTH University Hospital Aachen, Aachen, Germany

**Keywords:** seizure, stroke, MRI, DWI, hippocampus

## Abstract

**Introduction:**

Magnet resonance imaging (MRI) is the imaging gold standard for the evaluation of suspected epileptic seizures but also indispensable for detecting cerebral ischemia, using diffusion-weighted imaging (DWI) sequences. DWI restrictions can also occur following an epileptic seizure, thus mimicking cerebral ischemia. Postictal DWI lesions typically cross vascular territories and are confined to the cortex. Here, we present four illustrative cases with the unusual finding of reversible punctate postictal hippocampal DWI lesions, reminiscent of transient global amnesia (TGA).

**Methods:**

Case 1 was identified during video-EEG examination. We consecutively screened our database for similar cases, identifying three additional cases (3 male/1 female, age range 53–78 years). The initial MRI was performed within 5 days, a follow-up MRI within 4.5 months. All patients received video-EEG-monitoring.

**Results:**

All cases were initially referred for a first epileptic seizure. The occurrence of punctate hippocampal DWI lesions prompted the diagnosis of ischemic stroke with acute-symptomatic seizures. None of the patients featured classical symptoms of stroke or TGA. Follow-up MRIs were normal, ruling out ischemic stroke. During subsequent video-EEG workup one patient was diagnosed with epilepsy, the other patients with a first unprovoked seizure.

**Conclusion:**

We postulate that punctate hippocampal DWI lesions can be postictal phenomenon. Recognizing this imaging finding is relevant for the therapeutic management, we encourage referring patients for video-EEG monitoring in case of unconclusive findings. Besides vasogenic oedema related to neuronal hyperactivity, venous compression could be a potential pathomechanism. Prospective postictal imaging studies could help to better understand and quantify punctate hippocampal DWI lesions.

## Introduction

Magnetic resonance imaging (MRI) is the gold standard imaging technique for diagnosing acute ischemic stroke and for evaluating the etiology of seizures. Whereas acute stroke can be easily identified by changes in diffusion-weighted imaging (DWI) sequences, the focus of MRI diagnostics in seizures is to identify possible structural brain lesions. However, seizures frequently also lead to the emergence of transient MRI abnormalities. Theses comprise besides DWI changes, FLAIR-hyperintensities and T1-hypointensities and can pose a diagnostic challenge in distinguishing them from stroke-related changes (1, 2).

The frequency of transient seizure-associated MRI abnormalities ranges between 8 and 41% (2, 3) and is dependent on the type of seizure. DWI changes are more prevalent in status epilepticus, seizure clusters, and focal seizures with impaired consciousness than in single bilateral tonic–clonic seizures or focal seizures with preserved consciousness (3–6). DWI changes are typically confined to the cortical and subcortical layer and the hippocampus and follow a gyral distribution. Notably, extensive MRI changes crossing vascular territories help differentiate these patterns from ischemic stroke.

The occurrence of a first unprovoked seizure usually prompts MRI evaluation. A careful interpretation of DWI-changes is crucial to distinguish changes related to unprovoked seizures from acute symptomatic seizures in the aftermath of ischemic stroke (7, 8).

In our cases series we describe an unusual form of circumscribed postictal DWI changes that had a punctate pattern and were limited to the hippocampal formation, reminiscent of DWI changes observed in transient global amnesia (TGA). We aim to highlight the diagnostic challenges and raise the awareness of punctate hippocampal DWI lesions as a postictal phenomenon.

## Methods

### Patient selection

The index patient (case 1) was identified during video-EEG monitoring workup. His referral diagnosis was an acute-symptomatic seizure following ischemic stroke, based on a punctate hippocampal DWI restriction. We then conducted an in-house database search to identify additional cases with similar DWI patterns. We specified the following inclusion and exclusion criteria:

- Initial 3-Tesla-MRI performed within 7 days of the seizure, including FLAIR, DWI/ADC, T2, and T1 sequences, and displaying a punctate hippocampal DWI restriction.
- Clinical presentation inconsistent with transient global amnesia (TGA).
- Follow-up 3-Tesla MRI (according to HARNESS-protocol and the guidelines of the German Neurological Society, with additional DWI/ADC) within 5 months of the initial seizure with absence of hippocampal DWI or FLAIR lesions.
- First EEG conducted within 7 days of the initial seizure.
- Secondary diagnostic workup with video-EEG monitoring.

Following the criteria, we identified five additional cases with similar DWI patterns. However, two cases were excluded because no follow-up MRI was available.

We extracted sex, age, patient history, seizure semiology, EEG, video-EEG and MRI findings from in-house medical records.

## Case presentations

The patients (three male, one female) were aged between 53 and 78 years. Three presented with bilateral tonic–clonic seizures, while one person displayed a focal seizure with impaired consciousness upon presentation. None had a prior history of seizures or significant comorbidities. None of the patients received antiseizure medication (ASM) prior to the first seizure. However, patient 4 was treated with levetiracetam for 1.5 days following the first seizure. Detailed clinical information is provided in Table 1.

**Table 1:** Demographic and clinical characteristics of the patients.

Case	Age, sex	Comorbidities	Reason for ER	MRI	EEG	Final diagnosis
1	58, m	none	First focal (cloni of right upper limb) to bilateral tonic–clonic seizure	Timeframe after seizure: 5 days Result: Punctate lesion left hippocampus Timeframe of follow-up MRI: 3.5 months	Resting state-EEG: Timeframe after seizure: 6 days Result: Mild focal slowing bitemporal EEG-Monitoring-Unit: Timeframe after seizure: 3.5 months Result: Normal	Unprovoked seizure w/o diagnosis of epilepsy
2	78, m	prostate cancer (stable disease)	First focal seizure with impaired consciousness (Confusion - > amnesia)	Timeframe after seizure: 2 days Result: Punctate lesion right hippocampus Timeframe of follow-up MRI: 4.5 months	Resting state-EEG: Timeframe after seizure: 4 days Result: Focal slowing bitemporal left > > right EEG-Monitoring-Unit: Timeframe after seizure: 1.5 months Result: Focal slowing bitemporal and temporal epileptic discharges on the left side	Focal, non-lesional epilepsy
3	67, f	arterial hypertension, migraine	First focal (autonomic: nausea) to bilateral tonic–clonic seizure	Timeframe after seizure: 3 days Result: Punctate lesion left hippocampus Timeframe of follow-up MRI: 1.5 months	Resting state-EEG: Timeframe after seizure: 2 days Result: Focal slowing bitemporal left > right EEG-Monitoring-Unit: Timeframe after seizure: 1.5 months Result: Focal slowing temporal left	Unprovoked seizure w/o diagnosis of epilepsy
4	53, m	arterial hypertension, obesity	First bilateral tonic–clonic seizure	Timeframe after seizure: 2 days Result: Multiple punctate lesions in both hippocampi Timeframe of follow-up MRI: 8 days	Resting state-EEG: Timeframe after seizure: 1 day Result: Normal EEG-Monitoring-Unit: Timeframe after seizure: 3.5 months Result: Normal	Unprovoked seizure w/o diagnosis of epilepsy

**Figure 1:**
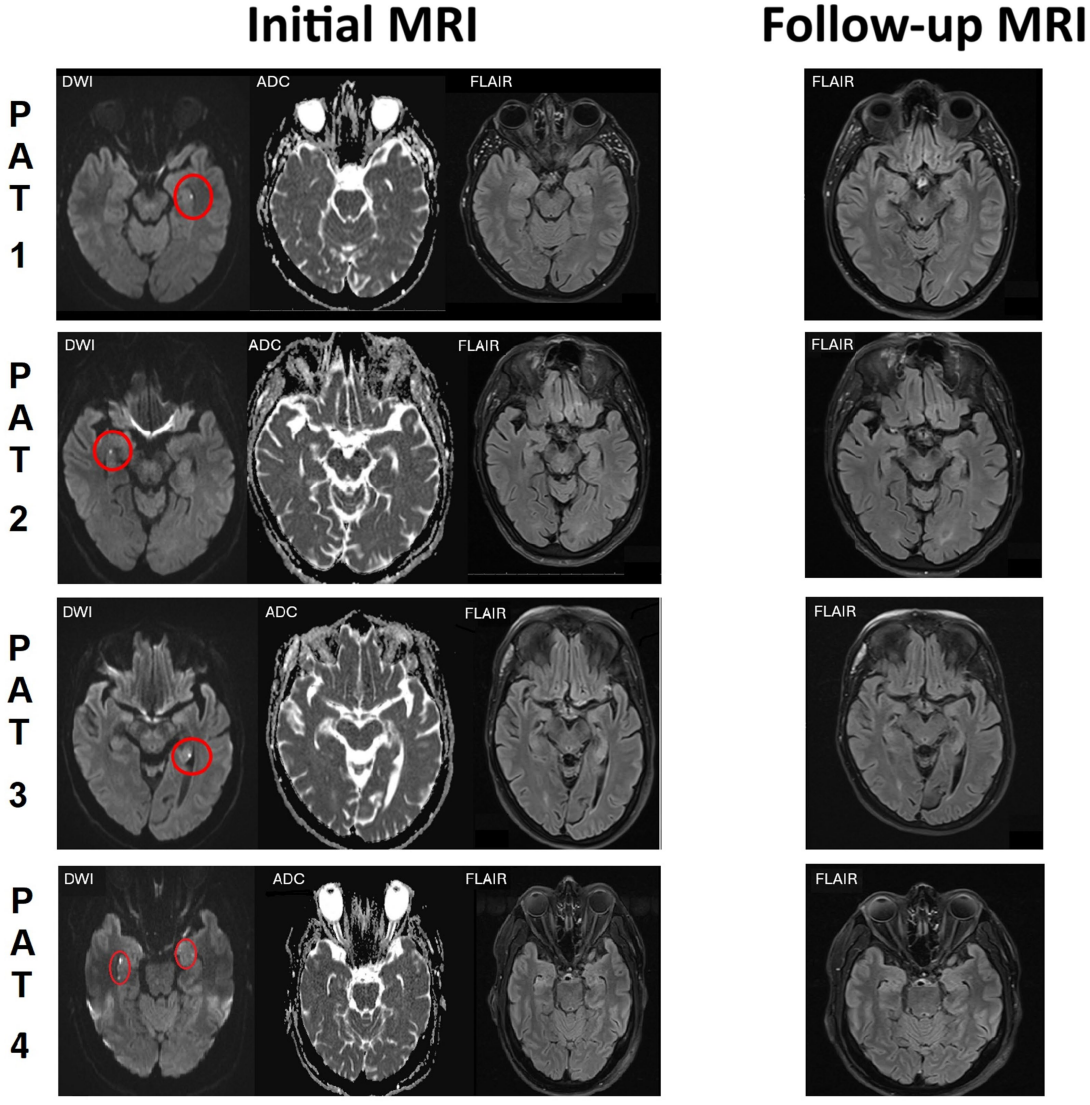
Initial MRI and follow-up MRI of the patients. Left column (initial imaging): DWI on the left, ADC-map in the middle, FLAIR-weighted image on the right. Punctate hippocampal DWI lesions in all 4 patients circled in red. Right column (follow-up imaging after 8 days - 3.5 months): FLAIR-weighted imaging.

The CT scan performed after emergency room (ER) admission was normal in all patients. Standard 10-20-EEG performed after one to six days after the first seizure showed no epileptic activity but regional temporal slowing in three patients. The 3 T-MRI was performed after two to five days after the initial seizure, displaying punctate hippocampal DWI lesions (left hippocampus in 2, right hippocampus in 1, and bilateral in 1 case) (Figure 1). The MRI findings prompted the diagnosis of ischemic stroke during the initial clinical workup. The seizures were interpreted as acute-symptomatic seizures. Of note, typical stroke symptoms, such as hemiparesis, hypaesthesia or speech disturbances, were absent and the neurological exams were normal. Moreover, symptoms concordant with TGA, such as short-term memory dysfunction, were also absent, although TGA was considered a potential differential diagnosis by the clinicians involved in primary workup due to the presence of the punctate hippocampal lesions.

All patients were referred to video-EEG monitoring between 1.5 to 3.5 months after the initial seizure. In one case (patient 2), frequent left temporal interictal epileptic discharges (IEDs) were recorded, prompting the diagnosis of focal epilepsy. The other patients showed no epileptiform EEG abnormalities during video-EEG monitoring. Follow-up MRIs revealed no residual DWI abnormalities or FLAIR lesions at the sites of the initial findings. For the three patients without epileptiform EEG abnormalities, the final diagnosis was changed to a singular unprovoked seizure.

## Discussion

We present four cases with an unusual postictal MRI pattern, featuring unilateral or bilateral punctate hippocampal DWI restrictions. These patterns deviate from MRI changes usually associated with seizures (9) and led to the initial misinterpretation of the cases. This series aims to raise the awareness of postictal punctate hippocampal DWI lesions and to prevent potentially unnecessary and harmful therapeutic interventions.

Unlike previously described postictal DWI alterations, we found a much more spatially constrained diffusion restriction (9). Moreover, postictal DWI abnormalities are frequently associated with convulsive or non-convulsive status epilepticus, while after single seizures their occurrence is less frequent (9). Unlike our cases, postictal DWI changes often exclusively affect cortical and subcortical layers and display an extensive gyral distribution, potentially across vascular territories (10, 11). Although the hippocampus is a known site of postictal MRI abnormalities, prior descriptions involve more expansive sections of the hippocampus and adjacent structures such as the amygdala or parahippocampal gyrus (2, 9, 10).

A common hypothesis of peri-ictal T2, FLAIR, and DWI changes is the occurrence of cytotoxic or vasogenic oedema caused by neuronal hyperexcitation, with failure of K^+^/Na^+^-ATPase function or breakdown of the blood–brain barrier (12, 13). Vasogenic oedema often presents as hyperintensity on apparent diffusion coefficient (ADC) maps, indicating transient and reversible changes. However, hypointense ADC signals in highly frequent seizures or refractory status epilepticus can signal permanent brain damage in the form of permanent cortical or subcortical cell loss, cortical laminar necrosis, or hippocampal sclerosis (6, 14). In recent years arterial-spin-labeling (ASL)-MRI-sequences were increasingly used to examine peri-ictal changes of cerebral perfusion, especially ictal hyperperfusion (4), but were not available in our cohort. ASL sequences are most helpful in status epilepticus or if an MRI-examination can be performed immediately after the occurrence of a seizure.

In our cases, all postictal DWI lesions were clearly punctate and limited to the hippocampus, suggesting alternative mechanisms. Given their similarity to DWI changes found in TGA that have been linked to physical exertion, similar pathophysiological mechanisms such as venous congestion with transient hippocampal hypoperfusion could potentially be involved (15). Hypothetically, the tonic phase of bilateral tonic–clonic seizures could give rise to prolonged venous congestion, which is also suggested as the cause of the so called ‘trout phenomenon’ (periorbital petechial hemorrhages) that is sometimes observed after tonic–clonic seizures (16). In focal seizures with impaired consciousness forced head version could also transiently compress the ipsilateral internal jugular vein, consecutively affecting venous drainage. Considering the fact that the direction of head version is normally in the opposite direction of the seizure onset side, this could also be an explanation, why the DWI lesion in our patient with left-sided focal epilepsy was located in the right hippocampus, even though there were no assured information on a head version in this patient. While ADC hypointensity in ischemic stroke or prolonged seizure-related hyperexcitation often indicates irreversible neuronal damage, it is not associated with permanent cell loss in TGA, as evidenced by its benign clinical course (17). Similarly, in all our cases initial hypointense signal ADC signals were absent in follow-up MRIs and did not result in persistent T2 lesions. Although our cases series cannot provide proof of related pathophysiologic patterns, the similarity to TGA DWI changes is striking and warrants further scientific attention.

Although according to literature, punctate postictal hippocampal DWI are observed rarely (15, 18), there might be unreported and unrecognized cases. For instance, in the case of unobserved seizures, the hippocampal MRI abnormalities could lead to mislabelling as ischemic stroke or TGA. Alternatively, as in our cases, the initial seizure was erroneously interpreted as acute-symptomatic seizure as a result of ischemic stroke. Undoubtedly, misinterpretation of DWI restrictions could prompt unnecessary medical treatments while overlooking the diagnosis of epilepsy. A correct diagnosis is also critical for socio-economic aspects such as fitness to drive a vehicle, work-related safety and its impact on society.

Our study is limited due to its retrospective nature and small sample size. Moreover, the descriptions of the initial seizures were based on reports from friends, family or other bystanders. To appreciate the real prevalence of punctate postictal hippocampal DWI lesions larger prospective cohort studies would be necessary, including ASL-sequences.

DWI lesions can pose a considerable diagnostic challenge. In the case of punctate hippocampal DWI lesions an ictal genesis should be considered and possibly corroborated by careful history-taking and neurological examination. In the absence of clinical evidence for ischemic stroke or TGA, video-EEG monitoring and a follow-up MRI can support the diagnostic workup. Besides vasogenic oedema related to neuronal hyperactivity, venous compression could be a potential pathomechanism. Prospective postictal imaging studies could help to better understand and quantify punctate hippocampal lesions.

## Data Availability

The datasets presented in this article are not readily available because of ethical and privacy restrictions. Requests to access the datasets should be directed to the corresponding author.
